# The effect of hip manipulation on muscle strength, proprioception and motor tests of basketball players during training

**DOI:** 10.3389/fbioe.2025.1632262

**Published:** 2025-08-28

**Authors:** Rafał Studnicki, Zuzanna Patrzykąt, Adam Kawczyński

**Affiliations:** ^1^ Department of Physiotherapy, Medical University of Gdańsk, Gdańsk, Poland; ^2^ Student Scientific Circle of Orthopaedic Manual Therapy, Medical University of Gdańsk, Gdańsk, Poland; ^3^ Department of Sport Didactics, Wroclaw University of Health and Sport Sciences, Wrocław, Poland

**Keywords:** diagonal manual therapy, lower limb stability, dynamic balance, gluteus medius activation, neuromuscular system

## Abstract

**Introduction:**

This study aimed to (i) investigate the effects of diagonal manual therapy on gluteus medius activation and (ii) examine its impact on quadriceps femoris activation, both critical for lower limb stability.

**Methods:**

Nine male basketball players (mean age 23.8 ± 4.1 years) participated in a randomized crossover trial, with measurements taken before the intervention, immediately after, and following a training session. Muscle activation of the gluteus medius, tensor fascia lata, and quadriceps was assessed via EMG, alongside evaluations of proprioception, the Y-Balance Test, and Standing Broad Jump. Repeated-measures ANOVA revealed significant time effects for all Y-Balance Test directions (anterior, posteromedial, and posterolateral) with large effect sizes (partial η^2^ > 0.70).

**Results:**

A significant interaction between intervention and assessment was also observed for proprioception at 90° hip flexion (p = 0.073), with a very large effect size (partial η^2^= 0.835). Main effects of the intervention were significant for maximal and mean activation of the gluteus medius, maximal activation of the tensor fascia lata, and maximal and mean activation of the vastus medialis, with the vastus lateralis showing especially notable maximal activation (partial η^2^ > 0.65). Furthermore, significant interactions with very large effect sizes (partial η^2^ often > 0.80) emerged for maximal activation of the gluteus medius, vastus lateralis (maximal, mean, and median values), rectus femoris maximal activation, and vastus medialis mean activation.

**Discussion:**

These findings provide preliminary evidence that diagonal manual therapy effectively enhances neuromuscular activation of the gluteus medius, a key muscle for lower limb stability. Moreover, the intervention influenced activation of other critical muscles such as the vastus lateralis and quadriceps femoris, suggesting a broader neuromuscular facilitation that may benefit dynamic postural control. Improvements in dynamic balance and proprioception further indicate potential functional gains.

## Introduction

Basketball is a highly dynamic and physically demanding sport, requiring athletes to possess excellent physical fitness and advanced motor skills. Due to the sport’s intense nature, injuries, particularly to the lower extremities, are common. Lower limb injuries account for approximately 63.7% of all basketball-related injuries, with ankle injuries comprising 21.9% and knee injuries 17.8% (Andreoli et al., 2018). Ankle sprains and ligament strains are especially prevalent, often resulting from rapid direction changes, jumping, landing, and ball contact (Aksović et al., 2024). Recent studies using data-driven deep learning models have provided new understandings into ligament fatigue failure mechanisms, emphasizing the high risk and complexity of ligamentous injuries in lower limbs during dynamic sports like basketball (Xu et al., 2025). Moreover, emerging research has optimized landing strategies that could significantly reduce the risk of lower limb injuries by improving neuromuscular control and biomechanical patterns (Xu et al., 2024). Upper limb injuries, particularly to the hands, fingers, and wrists, represent 12%–14% of total injuries (Andreoli et al., 2018). Injury patterns vary by age, skill level, and gender. For instance, children and adolescents are more prone to head injuries, while males tend to experience a higher injury rate than females (Andreoli et al., 2018; Hammig et al., 2007). The player’s position on the court also influences injury risk, with shooting guards sustaining the highest percentage of injuries (47.8%), followed by centers (34.8%) and point guards (17.4%) (Aksović et al., 2024). Despite various preventive and rehabilitative interventions focusing on proprioception, balance, and neuromuscular control, injury rates remain a significant concern in basketball. This raises the question of whether current approaches fully address the complex neuromuscular mechanisms involved in injury prevention.

Although systematic proprioceptive and balance training programs have demonstrated reductions in injury incidence (de Vasconcelos et al., 2018; Riva et al., 2016), several limitations in the existing literature remain. Most studies focus on generalized training protocols and overlook the integration of manual therapy techniques specifically targeting critical stabilizing muscles. Notably, the effects of manual therapy, such as diagonal hip manipulation, on muscle activation–particularly of the gluteus medius, a key hip stabilizer–have not been sufficiently investigated. Additionally, while proprioception assessments are common, many fail to incorporate direct measures of neuromuscular activation through tools like surface electromyography (sEMG), limiting understanding of underlying mechanisms.

Impaired balance and proprioception are significant risk factors for injuries in basketball, particularly ankle sprains, knee injuries, and low back pain (Riva et al., 2016). Athletes with compromised proprioception are up to seven times more likely to sustain injuries compared to those with adequate neuromuscular control. Such injuries can sideline players for several weeks and contribute to the weakening of other stabilizing structures in the lower limbs. Proprioceptive deficits, in particular, have been identified as predictors of ankle injuries in collegiate basketball players (Payne et al., 1997). While several effective interventions have been demonstrated, such as systematic proprioceptive and balance training programs showing an 81% reduction in ankle sprains over 6 years (e.g., 4), there remains a need to explore and integrate multiple complementary approaches to optimize injury prevention. Balance is not only vital for injury prevention but also fundamental for core basketball skills such as passing, shooting, and dribbling (Nikolaos et al., 2012). Evidence also suggests that postural balance training significantly improves Functional Movement Screen (FMS) scores, outperforming general physical training in reducing injury risk (Zhang and Wu, 2023). Complementary preventive strategies include a properly structured preparation period and warm-up routines aimed at enhancing range of motion (ROM) and balance, particularly in the ankles. Kinesiotaping, bracing, and appropriate footwear have also been shown to contribute to injury prevention (Padua et al., 2019). Moreover, manual therapy has demonstrated positive effects, such as pain reduction and increased dorsiflexion, and when combined with exercise, it lowers the likelihood of injury recurrence (de Ruvo et al., 2022).

Proprioception plays a crucial role in basketball performance and injury prevention, with studies showing that deficits in joint position sense (JPS) and postural control contribute to functional instability and recurrent injuries. Assessment tools such as the Y-Balance Test have demonstrated improvements in lower limb stability following training (Benis et al., 2016), while JPS tests help identify proprioceptive impairments (Takasaki et al., 2020), and the Standing Broad Jump is used to evaluate lower limb strength and asymmetries (Pérez-Castilla et al., 2021). Importantly, proprioception relies heavily on effective neuromuscular control, which involves the coordinated activation of muscles to maintain joint stability. However, most current assessments overlook direct evaluation of muscle activity, which is essential for understanding neuromuscular function related to proprioception and injury prevention. In this regard, surface electromyography (sEMG) offers a promising method for assessing muscle activation patterns and neuromuscular coordination in athletes (Studnicki et al., 2024a). Research supports the integration of proprioceptive training in basketball: a 6-year study found that systematic proprioceptive programs significantly reduced ankle, knee, and low back injuries (Riva et al., 2016) and a randomized controlled trial showed that multistation proprioceptive exercises reduced ankle injury risk by 65% while improving balance and joint position sense (Eilis et al., 2010). Additionally, players with a history of repeated ankle sprains display increased postural sway and repositioning errors (Fu and Hui-Chan, 2005), and proprioceptive acuity has been linked to skill execution, with wrist and elbow JPS correlating positively with free-throw accuracy (Sevrez and Bourdin, 2015).

Currently, there is a lack of studies in the literature specifically evaluating the effects of manual therapy–particularly diagonal manipulation–on the activation of the gluteus medius muscle, which is a primary stabilizer of the hip during multiplanar movements. The hypothesis that diagonal manipulation would specifically target the gluteus medius is based on its anatomical and functional role as a primary stabilizer of the hip during multiplanar movements. This technique involves joint positioning and mobilizations along diagonal planes that closely mimic functional hip patterns, potentially enhancing proprioceptive feedback and neuromuscular activation of the gluteus medius more effectively than single-plane interventions. Therefore, the present study aimed to investigate whether diagonal hip manipulation can enhance activation of the gluteus medius and quadriceps femoris muscles, as well as improve dynamic balance and proprioception in basketball players. We hypothesize that this specific manipulation technique will provide greater neuromuscular stimulation of the gluteus medius compared to traditional interventions, potentially contributing to injury prevention and improved athletic performance.

## Materials and methods

The Independent Bioethics Committee for Scientific Research at the Medical University of Gdańsk approved this study on 03 March 2025 (Resolution No. KB/95/2025). Additionally, the study was registered in Clinical Trials: NCT06901089.

Participants were thoroughly informed about the study, including a simplified overview of the protocol. They provided written informed consent prior to participation, acknowledging their voluntary involvement and their right to withdraw from the study at any time without any consequences. The study followed the ethical guidelines set forth in the Declaration of Helsinki.

### Participants

Twelve basketball men of the First Basketball League in Poland were invited to participate in this study, but three players of them were excluded: one was not 18 years old and two had an injury. The study was completed by nine players. To participate in the study, participants had to meet the following criteria: (i) actively play in the first league of basketball (ii) be in good health without a recent history of injury or illness; (iii) be at least 18 years old; and (iv) engage in all phases of the study. The exclusion criteria were: (i) suffered injury or illness during the examination; (ii) suffered an injury to the lower limb (thigh, lower leg, or foot) in the last 6 months, pain in these joints, excessive mobility of the lower limb joint, or any neurological or connective tissue disease.

Given the study’s aim to evaluate intervention effects in a consistent sport context, all participants were recruited from a single professional basketball team in Poland’s First Basketball League. This ensured homogeneity in training, coaching, and competition level, reducing external variability and bias. Although the sample was limited to nine athletes, the within-subject crossover design increased statistical power by using each participant as their own control. Additionally, these elite athletes enhance the ecological validity of the results for similar performance settings.

### Study design

**FIGURE 1:**
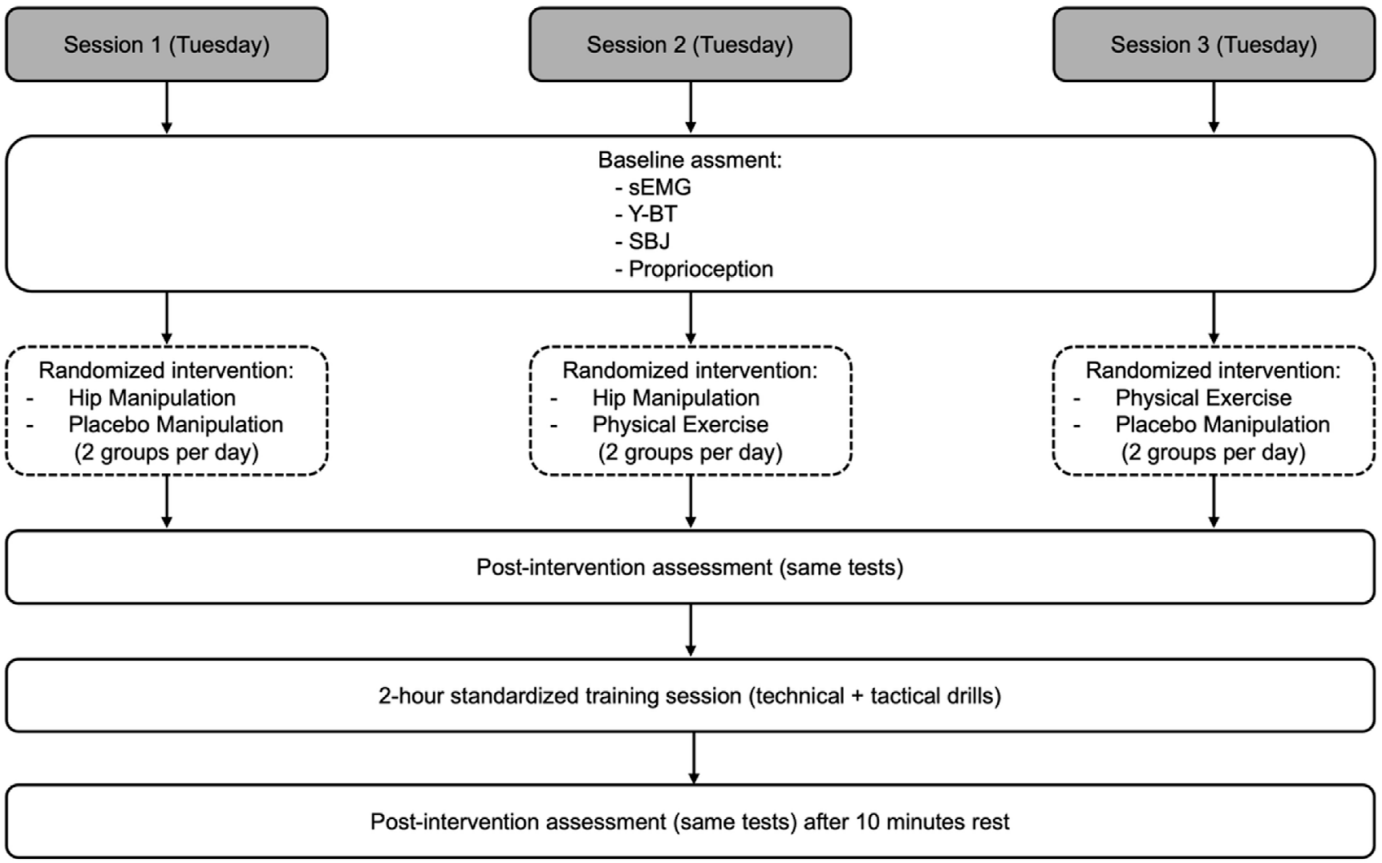
Experimental design showing the three-session crossover protocol, timing of assessments, and randomized interventions. Each session included three measurement time points and a standardized basketball training. All players completed all three interventions.

This study was a randomized, crossover experimental study, consisting of three separate experimental sessions per participant, conducted over a 3-week period. Each player participated in three different intervention conditions (hip manipulation, placebo manipulation (Control group), and forward lunge exercise). Measurements were conducted at three time points in each session: (1) baseline (before intervention), (2) immediately after the intervention, and (3) after a standardized 2-h basketball training session. The EMG signal was recorded from the gluteus medius, tensor fascia lata, and quadriceps (lateral head, medial head, and rectus femoris). Proprioception, the Y-Balance Test (Y-BT), and the Standing Broad Jump (SBJ) were also evaluated (Figure 1).

### Setting

A double-blind procedure was implemented in this study, whereby both the operator administering the intervention and the participants were blinded to the specific intervention being applied during each session, minimizing potential bias. The randomized assignment of intervention order further reduced expectancy effects.

The study was carried out at the end of March and beginning of April 2025, involving three separate experimental sessions per participant. Each session was conducted individually in the afternoon (at 16:00).

Upon arrival at the research facility, each participant underwent an initial evaluation, which included measurements of body weight, height, and dominant lower limb length. This was followed by a standardized 15-min warm-up, consisting of 5 min of jogging, 5 min of stretching exercises for the lower limbs, and 5 min of sport-specific drills. The pace and intensity of the jogging and stretching were self-selected by the participants, reflecting their usual warm-up routines. After a short rest period of 3 min, the baseline assessments were conducted. These included surface electromyography (sEMG) recordings, the Y-Balance Test (Y-BT), the Standing Broad Jump (SBJ), and a proprioception test. The players were randomly assigned to different testing sequences to minimize order effects. Immediately after the baseline assessments, participants received one of three possible interventions: (i) hip joint manipulation, (ii) placebo manipulation (involving manual contact without therapeutic intent–Control group), or (iii) a standardized forward lunge exercise. The selection of the intervention for each session followed a randomized protocol within a partial crossover design. While only two of the three intervention conditions (manipulation, control, or exercise) were applied per day, each participant completed all three experimental conditions across the three sessions.

Following the intervention, the same four performance tests (sEMG, Y-BT, SBJ, and proprioception) were repeated to assess immediate post-intervention effects. After this, participants engaged in a 2-h basketball training session led by the team’s coach. This session was standardized and identical across all study days, incorporating typical technical, tactical, and game-based drills regularly used by the team. The aim of including this training session was to simulate real-world physical load and evaluate performance outcomes in a post-fatigue context. After a 10-min rest period following training, a final set of measurements was collected using the same procedures and order as before. All assessments were conducted on Tuesdays to ensure consistency in scheduling, environment, and training content.

The interventions were structured across three consecutive Tuesdays as follows: on Day 1, Group A received hip manipulation, and Group B received the placebo manipulation; on Day 2, Group A again received manipulation, and Group C received the exercise intervention; on Day 3, Group B received the placebo manipulation, and Group C received the exercise. All participants completed all three experimental conditions (manipulation, control, and exercise) across the study; however, on each testing day, only two of the three interventions were administered to different groups. This scheduling ensured a balanced evaluation of each intervention while minimizing fatigue and carryover effects.

### Intervention

Muscle fatigue assessments focused on the dominant limb and were based on changes in neuromuscular activation and functional performance across the session timeline. After completing a standardized, investigator-led warm-up protocol–including 10 min of dynamic lower extremity stretching and 5 min of isometric lower extremity exercises–participants completed baseline assessments. Assessments were performed by two investigators: one specialized in EMG equipment and the other in physical performance assessments.

During the baseline (pre-training) evaluation, participants were introduced to the testing equipment and procedures. sEMG was recorded from the gluteus medius, tensor fascia lata, and quadriceps (rectus femoris, vastus lateralis, and vastus medialis). Each muscle was assessed three times during specific movement phases (Padua et al., 2019).

### Hip manipulation

**FIGURE 2:**
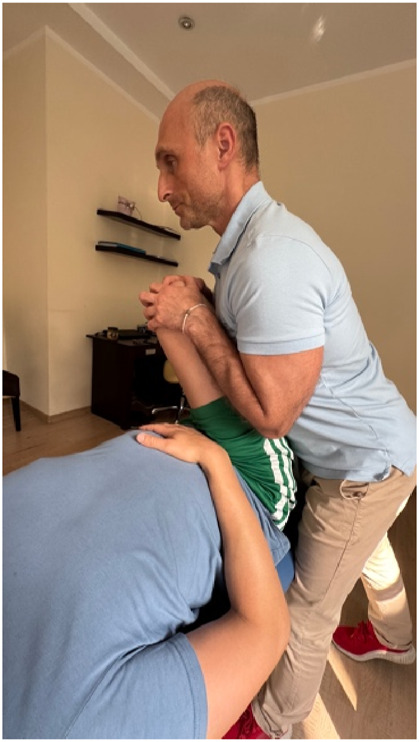
Hip manipulation technique.

Participants in the intervention group received a lateral, dorsal, and caudal diagonal manipulation with a 2-s high-velocity, low-amplitude (HVLA) short-duration tension. Participants were briefed on the procedure. Each participant lay in a supine position with maximal hip flexion and adduction, and additional external rotation at the hip joint (the volunteer’s knee was directed toward the opposite shoulder) was added to further lengthen the muscle fibers (Studnicki et al., 2024b). The therapist positioned himself on the caudal-lateral side of the participant, placing both hands around the knee joint from above, with the therapist’s anterior part of the torso in as close contact with the lateral part of the thigh as possible (Figure 2). The therapist then moved the thigh, moving it obliquely along the femur in the following directions: lateral, dorsal, caudal, until maximal tension was achieved in the hip joint. From this point, the time was measured for 2 s. At this point, the therapist made sure that the participant felt no discomfort and then performed a 2-s oblique manipulation. This technique was performed once for each participant. Each manipulation was performed by a physiotherapist specializing in manual therapy with 25 years of experience.

### Placebo manipulation

The participants in the control group received a sham intervention. The procedure mirrored the HVLA technique but without inducing tissue tension during the oblique movement of the thigh (Studnicki et al., 2025). Once the desired position was achieved, the therapist did not measure the 2-s tension, preventing an increase in tissue tension in the hip joint. The therapist then made sure that the participant felt no discomfort and performed a movement that mimicked the manipulation without increasing tissue tension.

### Exercise forward lunge with a long step

Each participant took part in a pre-test session and the experimental conditions were discussed. Given their competitive level, all participants were already familiar with the forward lunge exercise and reported regularly performing it with dumbbells during training. The forward lunge was chosen as an active, sport-specific intervention to stimulate the same muscle groups assessed in the study (gluteus medius, quadriceps, and tensor fascia lata) and to serve as a functional comparator to the passive (manipulation) and Control interventions. The long-step variation promotes significant activation of the hip joint and surrounding musculature, providing proprioceptive and neuromuscular input similar in scope–but different in mechanism–to joint manipulation. Its relevance in injury prevention and neuromuscular conditioning in basketball contexts further justified its inclusion. Participants selected their habitual training load, typically ranging from 10 to 16 kg per hand, to ensure ecological validity and safety. During the exercise, the stride length was adjusted to maintain the shin bone approximately vertical at the bottom of the movement, keeping the knee over the foot. The trunk was kept in the habitual training posture. Participants began from a standing position, lunged forward, and returned to the starting position, with the same procedure applied for all (Escamilla et al., 2025).

### Measurements

#### Surface electromyography

Surface EMG data were collected for gluteus medius, tensor fascia lata, vastus medialis, vastus lateralis, rectus femoris during the maximal phase of voluntary isometric contraction (3 s recorded) (Naderza et al., 2024). SEMG data were collected and differentially amplified with a gain of 500 using TeleMyo DTS (Noraxon, Scottsdale, AZ, United States) with 1 cm^2^ Ag/AgCl surface electrodes (Sorimex, Toruń, Poland). Bandpass filtering was applied to the SEMG signals with cutoff frequencies between 15 and 500 Hz (Butterworth, 4th order), and data were sampled at 1,500 Hz (16-bit resolution) using an analog-to-digital converter. Bandpass filtering was applied to the SEMG signals (15–500 Hz) and sampled at 1,500 Hz (16-bit resolution) using an analog-to-digital converter. SEMG data were then archived and further processed using MyoResearch 2.8 software (Noraxon). Electrode placement and skin preparation, including shaving, abrasion, and alcohol cleaning, were according to SENIAM recommendations. Signal processing included full-wave rectification followed by smoothing using the root mean square (RMS) method with a moving time window of 300 ms. To allow comparison across participants and conditions, EMG amplitude values were normalized to the maximum voluntary isometric contraction (MVC) obtained during standardized maximal contractions. The normalized parameters analyzed included mean and maximum EMGRMS amplitude (expressed as a percentage of MVC) and the median frequency of the raw power spectrum of the SEMG signal (EMGMED, in Hz).

#### Y-balance test

The YBT was conducted using a reliable, standardized testing protocol as established by Plisky et al. (2009). The test was conducted in footwear that was designed specifically for the athlete in that discipline so as not to interfere with accurate reproduction of technical basketball movements (Benis et al., 2016). Participants were fully familiarized with all testing procedures through a practice trial conducted prior to the baseline measurements, in order to reduce potential learning effects and improve measurement reliability. Participants were fully familiarized with the testing procedures during the first trial. After the players performed a trial to familiarize themselves with the procedure.

To assess the YBT, we used a YBT setup consisting of a support platform to which 3 pieces of polyvinyl chloride tubing were attached in the reaching directions A, PM, and PL. Each tubing was marked at 5 mm intervals. The participant was required to reach with the opposite leg in the A, PM, and PL directions and push the indicator along the tubing that standardized the reach distance. Each player underwent three trials assessing the dominant limb and the mean of those three trials were considered. The distance achieved was measured from the most distal aspect of the reaching foot in the stance direction to the most distal aspect of the reaching foot in the A, PM, and PL directions.

#### Standing broad jump

Subjects began in a comfortable bilateral stance with each leg fully extended with the feet hip-width apart and the hands placed on the hips. The subjects were then instructed to jump forward as far as possible and land on both feet after countermoveing to a depth of their choosing. The subjects were instructed to jump and land on both feet simultaneously (Pérez-Castilla et al., 2021). The supervising experimenter asked the subjects to repeat the test three times after 1 min of rest in case the jump did not comply with these instructions. The best jump was considered for analysis.

#### Proprioception

Proprioception assessment was performed at the hip joint in random order for 30°, 45°, 90° flexion using a Saunders^®^ digital inclinometer (The Saunders Group, Inc. Chaska, MN, United States) that was attached to the athlete’s knee joint with tape. The assessment was performed in a supine position with the lower limb stretched on a couch and the upper limbs along the body. To ensure optimal performance, each participant was blindfolded during the test (Motealleh et al., 2016). Each assessment in one of the three positions was performed three times. At the starting position, the goniometer was zeroed, and the target angle was verbally communicated to the athlete. The athlete focused on a specific angle and maintained the flexed hip in this position for 5 s to fully perceive the target angle. The athlete was then asked to position the hip at the target angle, and the measurements were repeated 3 times for each target angle, and the angular errors of each measurement were averaged. The test was applied to the dominant limb.

#### Statistical procedures

Prior to conducting the main analysis, the normality of the distribution of each dependent variable across the three conditions was assessed using the Shapiro–Wilk test and confirmed by visual inspection of Q–Q plots. Despite the small sample size (n = 9), the assumption of normality was considered reasonably met. The assumption of sphericity was evaluated using Mauchly’s test. In cases where this assumption was violated (p < 0.05), the Greenhouse-Geisser correction was applied to adjust the degrees of freedom. In addition, due to the small sample size and the increased robustness of multivariate methods, the results Wilks’ Lambda were also reported and considered for interpretation. Given the crossover design, in which all participants were exposed to each of the experimental conditions, a repeated-measures ANOVA was conducted to examine the main effects of Condition (manipulation, exercise and control) and Time (assessment at the beginning, after the intervention and after the training session), as well as their interaction (Condition × Time). As the design was fully within-subjects, each participant served as their own control. When significant main or interaction effects were found, pairwise comparisons with Bonferroni adjustment were conducted to identify where the differences occurred. Effect sizes (e.g., partial eta-squared, η^2^_p_) were reported where relevant. All statistical analyses were performed using IBM SPSS Statistics (version 29.0). The significance level was set at p < 0.05.

## Results

The sample consisted of nine participants, with a mean age of 23.8 ± 4.1 years, mean body mass of 91.9 ± 11.9 kg, and mean height of 194.6 ± 8.8 cm.

**FIGURE 3:**
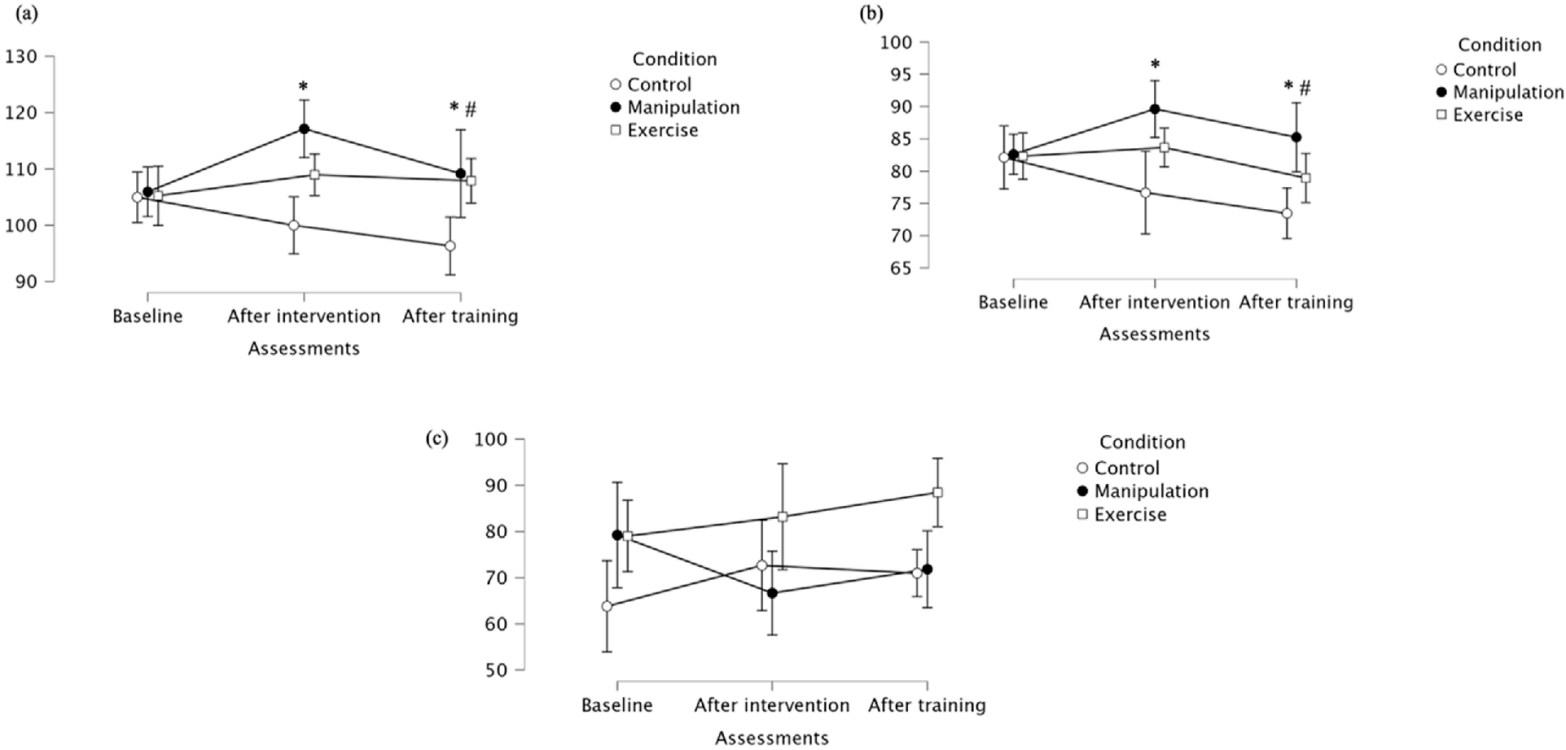
**(a)** Maximal; **(b)** Mean **(c)** Median activation of the gluteus medius across groups. * Indicates significant differences between control and manipulation conditions; # Indicates significant differences between manipulation and exercise conditions.

For gluteus medius maximal activation, there was a trend toward a main effect of assessment (p = 0.056), a significant main effect of condition (p = 0.004), and a significant assessment × condition interaction (p = 0.010), indicating that intervention type and its interaction with time influenced muscle activation (see Table 1 for ANOVA results). Post hoc tests revealed no baseline differences between groups. After intervention, the manipulation group showed significantly higher maximal activation than the Control group, with no significant differences compared to the Exercise group. Following training, the manipulation group maintained superior activation levels, significantly higher than both the Control and Exercise groups. No significant differences were found between Control and Exercise at any time point. Detailed descriptive data and specific pairwise comparisons are presented in Table 2. For gluteus medius mean activation, significant main effects were found for assessment (p = 0.012) and condition (p = 0.005), with a trend toward an interaction effect (p = 0.065), suggesting overall improvements over time and between groups, with possible interaction dynamics (Table 1). At baseline, no significant differences were observed between groups. After the intervention, the Manipulation group showed significantly higher mean activation than the Control group, and the Exercise group also improved, but without statistically significant differences compared to either group. Following training, the manipulation group maintained elevated activation, remaining significantly higher than the Control group, though the difference with the Exercise group did not reach statistical significance. For gluteus medius mean activation, significant main effects were found for assessment (p = 0.012) and condition (p = 0.005), with a trend toward an interaction effect (p = 0.065), suggesting overall improvements over time and between groups, with possible interaction dynamics (Table 1). At baseline, no significant differences were observed between groups. After the intervention, the Manipulation group showed significantly higher mean activation than the Control group, and the Exercise group also improved, but without statistically significant differences compared to either group. Following training, the manipulation group maintained elevated activation, remaining significantly higher than the Control group, though the difference with the Exercise group did not reach statistical significance. Gluteus medius median values showed no significant effect of assessment, but a significant main effect of condition (p = 0.028), indicating overall differences between groups regardless of time (Table 1). At baseline, median activation was relatively similar across groups. After the intervention, both the Manipulation and Exercise groups maintained comparable levels, while the Control group showed an increase. Post-training, however, the manipulation group exhibited a marked increase in median activation, significantly higher than the Exercise group, while the Control group remained stable. These findings suggest a delayed but meaningful benefit of the manipulation intervention on gluteus medius median activation, particularly after the training period. For all detailed median activation values. For detailed values, see Table 2 and Figure 3.

**TABLE 1:** Results of the repeated measures multivariate analysis (ANOVA repeated measures) for the effects of the intervention, assessment time, and their interaction on surface EMG data collected for gluteus medius, tensor fascia lata, vastus medialis, vastus lateralis, rectus femoris.

Dependent variable	Effect	Wilks’ Lambda	F	df (hyp, error)	*p*	Partial η^2^
Gluteus medius maximal value	Intervention	0.439	4.481	(2,7)	0.056	0.561
	Assessment	0.208	13.290	(2,7)	**0.004**	0.792
	Intervention x Assessment	0.099	11.427	(4,5)	**0.010**	0.901
Gluteus medius mean value	Intervention	0.281	8.942	(2,7)	**0.012**	0.719
	Assessment	0.225	12.033	(2,7)	**0.005**	0.775
	Intervention x Assessment	0.218	4.492	(4,5)	0.065	0.782
Gluteus medius median value	Intervention	0.841	0.660	(2,7)	0.546	0.159
	Assessment	0.360	6.219	(2,7)	**0.028**	0.640
	Intervention x Assessment	0.342	2.404	(4,5)	0.181	0.658
Tensor fascia lata maximal value	Intervention	0.345	6.646	(2,7)	**0.024**	0.655
	Assessment	0.470	3.944	(2,7)	**0.071**	0.530
	Intervention x Assessment	0.656	0.656	(4,5)	0.648	0.344
Tensor fascia lata mean value	Intervention	0.614	2.199	(2,7)	0.182	0.386
	Assessment	0.999	0.003	(2,7)	0.997	0.001
	Intervention x Assessment	0.522	1.145	(4,5)	0.432	0.478
Tensor fascia lata median value	Intervention	0.960	0.145	(2,7)	0.867	0.040
	Assessment	0.430	4.645	(2,7)	0.052	0.570
	Intervention x Assessment	0.337	2.457	(4,5)	0.175	0.663
Vastus medialis maximal value	Intervention	0.309	7.841	(2,7)	**0.016**	0.691
	Assessment	0.287	8.969	(2,7)	**0.013**	0.713
	Intervention x Assessment	0.240	3.949	(4,5)	0.082	0.760
Vastus medialis mean value	Intervention	0.299	8.192	(2,7)	**0.015**	0.701
	Assessment	0.634	2.024	(2,7)	0.203	0.366
	Intervention x Assessment	0.103	10.876	(4,5)	**0.011**	0.897
Vastus medialis median value	Intervention	0.963	0.134	(2,7)	0.877	0.037
	Assessment	0.758	1.117	(2,7)	0.379	0.242
	Intervention x Assessment	0.349	2.332	(4,5)	0.189	0.651
Vastus lateralis maximal value	Intervention	0.123	24.855	(2,7)	**<0.001**	0.877
	Assessment	0.172	16.886	(2,7)	**0.002**	0.828
	Intervention x Assessment	0.132	8.186	(4,5)	**0.020**	0.868
Vastus lateralis mean value	Intervention	0.696	1.529	(2,7)	0.281	0.304
	Assessment	0.489	3.651	(2,7)	0.082	0.511
	Intervention x Assessment	0.018	66.664	(4,5)	**<0.001**	0.982
Vastus lateralis median value	Intervention	0.778	1.001	(2,7)	0.415	0.222
	Assessment	0.178	16.207	(2,7)	**0.002**	0.822
	Intervention x Assessment	0.159	6.596	(4,5)	**0.031**	0.841
Rectus femoris maximal value	Intervention	0.447	4.337	(2,7)	0.060	0.553
	Assessment	0.777	1.002	(2,7)	0.414	0.223
	Intervention x Assessment	0.124	8.803	(4,5)	**0.017**	0.876
Rectus femoris mean value	Intervention	0.581	2.527	(2,7)	0.149	0.419
	Assessment	0.380	5.702	(2,7)	**0.034**	0.620
	Intervention x Assessment	0.247	3.805	(4,5)	0.088	0.753
Rectus femoris median value	Intervention	0.495	3.567	(2,7)	0.085	0.505
	Assessment	0.672	1.709	(2,7)	0.249	0.328
	Intervention x Assessment	0.486	1.321	(4,5)	0.376	0.514

Wilks’ Lambda, F-values, degrees of freedom, significance (p), and effect size (partial η^2^) are reported.

Bold values denote statistically significant results for Wilks’ Lambda, F(df_h_, df_e_), p, and partial η^2^ (p < 0.05).

**TABLE 2:** Detailed comparison of muscle activation and physical performance between groups over time.

Variable (unit)	Group	Baseline	Post-intervention	Post-training	Significant post-intervention comparisons	Significant post-training comparisons
Gluteus Medius						
Maximal activation (%)	Control	104.97 ± 2.56	105.94 ± 3.73	105.24 ± 5.10		
	Manipulation	99.98 ± 9.52	117.11 ± 6.69	108.96 ± 6.25	Manipulation > Control (p = 0.005)	Manipulation > Control (p = 0.009)
	Exercise	96.32 ± 8.24	109.17 ± 11.18	107.89 ± 6.04		Manipulation > Exercise (p = 0.009)
Mean activation (%)	Control	82.12 ± 6.44	82.62 ± 4.01	82.32 ± 3.33		
	Manipulation	76.67 ± 9.88	89.62 ± 5.90	83.66 ± 5.59	Manipulation > Control (p = 0.012)	Manipulation > Control (p = 0.005)
	Exercise	73.48 ± 5.97	85.25 ± 6.93	78.95 ± 5.85		
Median activation (%)	Control	63.79 ± 14.83	79.24 ± 20.20	79.01 ± 20.82		
	Manipulation	72.66 ± 13.29	66.65 ± 10.31	83.18 ± 18.15		Manipulation < Exercise (p = 0.038)
	Exercise	70.96 ± 10.10	71.82 ± 16.58	88.45 ± 14.66		
Tensor fascia lata						
Maximal activation (%)	Control	113.99 ± 9.62	106.32 ± 8.63	111.66 ± 7.64		
	Manipulation	111.54 ± 8.36	100.37 ± 7.41	106.08 ± 9.96	Manipulation < Control (p = 0.038)	
	Exercise	115.71 ± 14.37	108.43 ± 5.45	106.91 ± 9.30		
Mean activation (%)	Control	79.84 ± 8.05	79.09 ± 7.57	77.77 ± 6.79		
	Manipulation	75.12 ± 11.26	79.30 ± 6.92	79.30 ± 6.92		
	Exercise	81.05 ± 9.11	79.16 ± 9.76	79.16 ± 9.76		
Median activation (%)	Control	67.54 ± 17.64	79.03 ± 26.22	75.44 ± 9.17	Exercise < Manipulation	Exercise < Manipulation
	Manipulation	70.32 ± 26.97	79.03 ± 26.22	75.44 ± 9.17	Exercise < Control	Exercise < Control
	Exercise	63.89 ± 17.69	72.66 ± 28.61	91.16 ± 18.28		
Vastus lateralis						
Maximal activation (%)	Control	104.70 ± 7.40	100.88 ± 9.19	95.10 ± 5.81		
	Manipulation	104.16 ± 7.64	112.72 ± 7.42	109.69 ± 5.46	Exercise > Control (p < 0.05)	Exercise > Control (p < 0.05)
	Exercise	109.05 ± 6.11	113.68 ± 3.76	109.69 ± 5.46		
Mean activation (%)	Control	76.14 ± 9.34	75.01 ± 6.06	68.93 ± 5.66		
	Manipulation	74.52 ± 11.50	85.15 ± 7.88	78.97 ± 9.59		
	Exercise	76.64 ± 8.10	81.97 ± 4.32	76.64 ± 5.86		
Median activation (%)	Control	73.21 ± 9.70	62.47 ± 14.49	67.46 ± 10.67		
	Manipulation	64.10 ± 3.81	66.87 ± 11.86	63.48 ± 6.51	Baseline: Exercise > Manipulation (p = 0.041)	
	Exercise	65.18 ± 18.41	73.46 ± 7.84	76.33 ± 10.48		
Vastus medialis						
Maximal activation (%)	Control	106.93 ± 5.61	112.22 ± 5.91	106.93 ± 5.61		
	Manipulation	111.58 ± 5.01	119.11 ± 3.82	111.58 ± 5.01	Exercise < Control (p < 0.05)	Exercise < Control (p < 0.05)
	Exercise	103.30 ± 4.91	111.68 ± 7.14	103.30 ± 4.91		
Mean activation (%)	Control	73.83 ± 7.09	78.38 ± 5.63	73.83 ± 7.09		
	Manipulation	75.54 ± 8.92	85.35 ± 5.88	75.54 ± 8.92		
	Exercise	71.94 ± 7.61	76.27 ± 7.47	71.94 ± 7.61		
Median activation (%)	Control	61.98 ± 12.13	74.57 ± 9.33	66.24 ± 5.08	Baseline: Control > Exercise (p = 0.041)	
	Manipulation	57.31 ± 3.16	57.31 ± 3.16	61.83 ± 6.43		
	Exercise	58.74 ± 0.20	58.74 ± 0.20	66.56 ± 11.32		
Rectus femoris						
Maximal activation (%)	Control	109.77 ± 3.84	111.27 ± 10.01	111.27 ± 10.01		
	Manipulation	103.06 ± 6.70	113.80 ± 4.47	113.80 ± 4.47	Manipulation > Control (p = 0.003)	
	Exercise	103.23 ± 8.35	95.77 ± 12.86	95.77 ± 12.86		
Mean activation (%)	Control	75.73 ± 9.03	75.73 ± 9.03	68.52 ± 9.38		
	Manipulation	84.45 ± 6.20	84.45 ± 6.20	80.53 ± 6.13	Manipulation > Control (p = 0.029)	Manipulation > Control
	Exercise	81.45 ± 6.65	81.45 ± 6.65	81.45 ± 6.65		Exercise > Control
Median activation (%)	Control	76.83 ± 11.26	76.83 ± 11.26	76.83 ± 11.26		
	Manipulation	80.19 ± 11.64	80.19 ± 11.64	81.23 ± 10.65	Exercise < Manipulation (p = 0.008)	
	Exercise	66.87 ± 9.56	66.87 ± 9.56	77.75 ± 8.11		
Y-balance anterior						
Performance (cm)	Control	65.45 ± 10.53	76.85 ± 6.44	72.30 ± 10.67		
	Manipulation	62.93 ± 8.74	81.48 ± 6.54	77.41 ± 8.87	Manipulation > Control (p = 0.044 - baseline), Manipulation > Control (Post-Intervention)	Manipulation > Control (Post-Training)
	Exercise	62.37 ± 8.56	79.52 ± 9.42	75.93 ± 11.77	Exercise > Control (Post-Intervention)	Exercise > Control (Post-Training)
Y-balance posteromedial						
Performance (cm)	Control	110.85 ± 13.10	122.44 ± 7.68	122.78 ± 4.95		
	Manipulation	105.89 ± 11.88	128.52 ± 9.48	125.33 ± 6.87	Manipulation > Control (Post-Intervention)	Manipulation > Control (Post-Training)
	Exercise	107.44 ± 10.10	125.41 ± 8.89	123.37 ± 7.45	Exercise > Control (Post-Intervention)	Exercise > Control (Post-Training)
Y-balance posterolateral						
Performance (cm)	Control	116.78 ± 17.72	131.07 ± 8.44	127.63 ± 8.49		
	Manipulation	111.41 ± 10.82	137.85 ± 9.71	131.93 ± 8.41	Manipulation > Control (Post-Intervention)	Manipulation > Control (Post-Training)
	Exercise	110.00 ± 8.29	135.33 ± 11.03	132.48 ± 6.61	Exercise > Control (Post-Intervention)	Exercise > Control (Post-Training)
Standing broad jump						
Performance (cm)	Control	228.00 ± 30.06	229.56 ± 24.59	218.56 ± 33.75		
	Manipulation	220.56 ± 36.25	245.22 ± 20.17	222.89 ± 31.58		
	Exercise	210.56 ± 32.32	239.11 ± 20.45	237.00 ± 17.77		
Proprioception at 30∘						
Error (^∘^)	Control	30.52 ± 1.46	29.26 ± 0.93	31.56 ± 2.62		
	Manipulation	29.74 ± 2.67	30.59 ± 1.35	32.59 ± 5.22	Baseline: Manipulation < Exercise (p = 0.027)	
	Exercise	31.22 ± 2.11	30.33 ± 2.19	30.63 ± 1.81		
Proprioception at 45^∘^						
Error (^∘^)	Control	44.78 ± 3.34	45.33 ± 1.41	46.07 ± 4.76		
	Manipulation	45.96 ± 4.02	45.11 ± 0.74	47.15 ± 2.66		
	Exercise	46.04 ± 3.80	45.26 ± 2.62	44.26 ± 2.43		
Proprioception at 90^∘^						
Error (^∘^)	Control	83.70 ± 3.68	87.00 ± 3.76	87.67 ± 5.17		
	Manipulation	81.71 ± 3.49	90.13 ± 2.73	91.54 ± 2.98	Manipulation > Control (Post-Intervention)	Manipulation > Control (Post-Training)
	Exercise	86.29 ± 2.81	90.54 ± 2.40	91.37 ± 2.91	Manipulation > Exercise (Post-Intervention)	Manipulation > Exercise (Post-Training)

Bold values denote significant F(df_h_, df_e_), p, and partial η^2^ values (p < 0.05).

**FIGURE 4:**
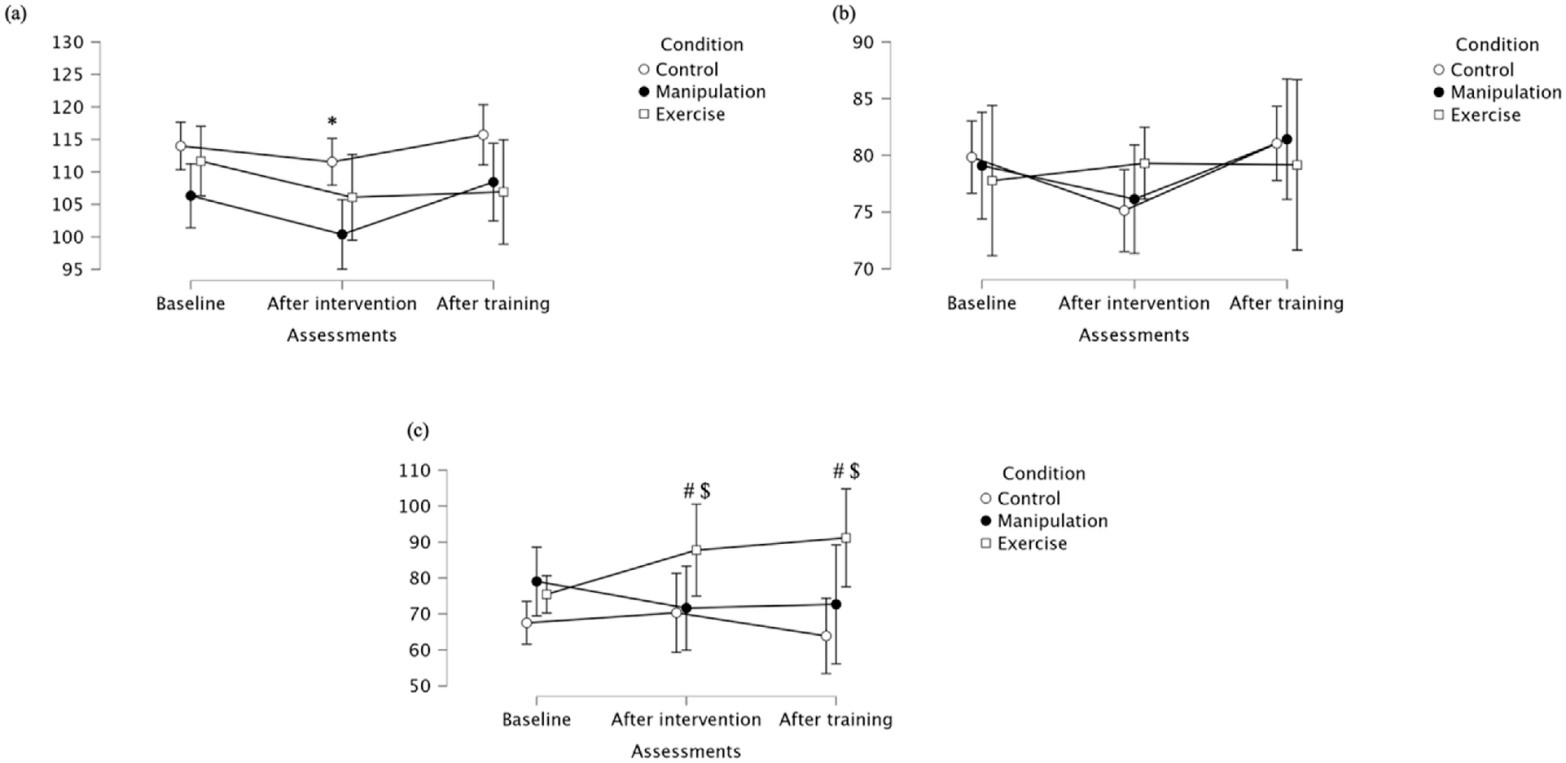
**(a)** Maximal; **(b)** Mean **(c)** Median activation of the tensor fascia lata across groups. * Indicates significant differences between control and manipulation conditions; # Indicates significant differences between manipulation and exercise conditions; $ Indicates significant differences between control and exercise conditions.

For tensor fascia lata maximal activation, a significant main effect of Assessment was observed (p = 0.024), indicating that activation levels changed over time. There was a trend toward a main effect of Condition (p = 0.071), but no significant interaction effect was found, suggesting that the pattern of change was similar across groups (Table 1). At baseline, the Control group showed the highest TFL maximal activation. After the intervention, a general reduction in maximal activation was observed across groups, with the manipulation group showing significantly lower activation than the Control group. Following the training period, activation values converged again, with no significant between-group differences at this stage. Regarding tensor fascia lata mean activation, no significant main effects of Assessment, Condition, or their interaction were found, indicating stable average activation levels throughout the study protocol (Table 1). Mean values were relatively consistent across groups and time points. These stable mean activation values contrast with the significant interaction found for median values, suggesting that while central tendency remained constant, distribution or variability in activation may have differed between groups and over time. Median values for tensor fascia lata revealed a significant interaction between Assessment and Condition (p = 0.041), with Exercise exercises showing lower median activation than Manipulation and Control after intervention and training (Table 1). At baseline, median activation values were more similar across groups. These results suggest that while mean activation levels remained stable, the distribution of activation levels–as reflected by median values–varied across groups over time, highlighting the differential impact of exercise modalities on tensor fascia lata muscle activation (Figure 4).

**FIGURE 5:**
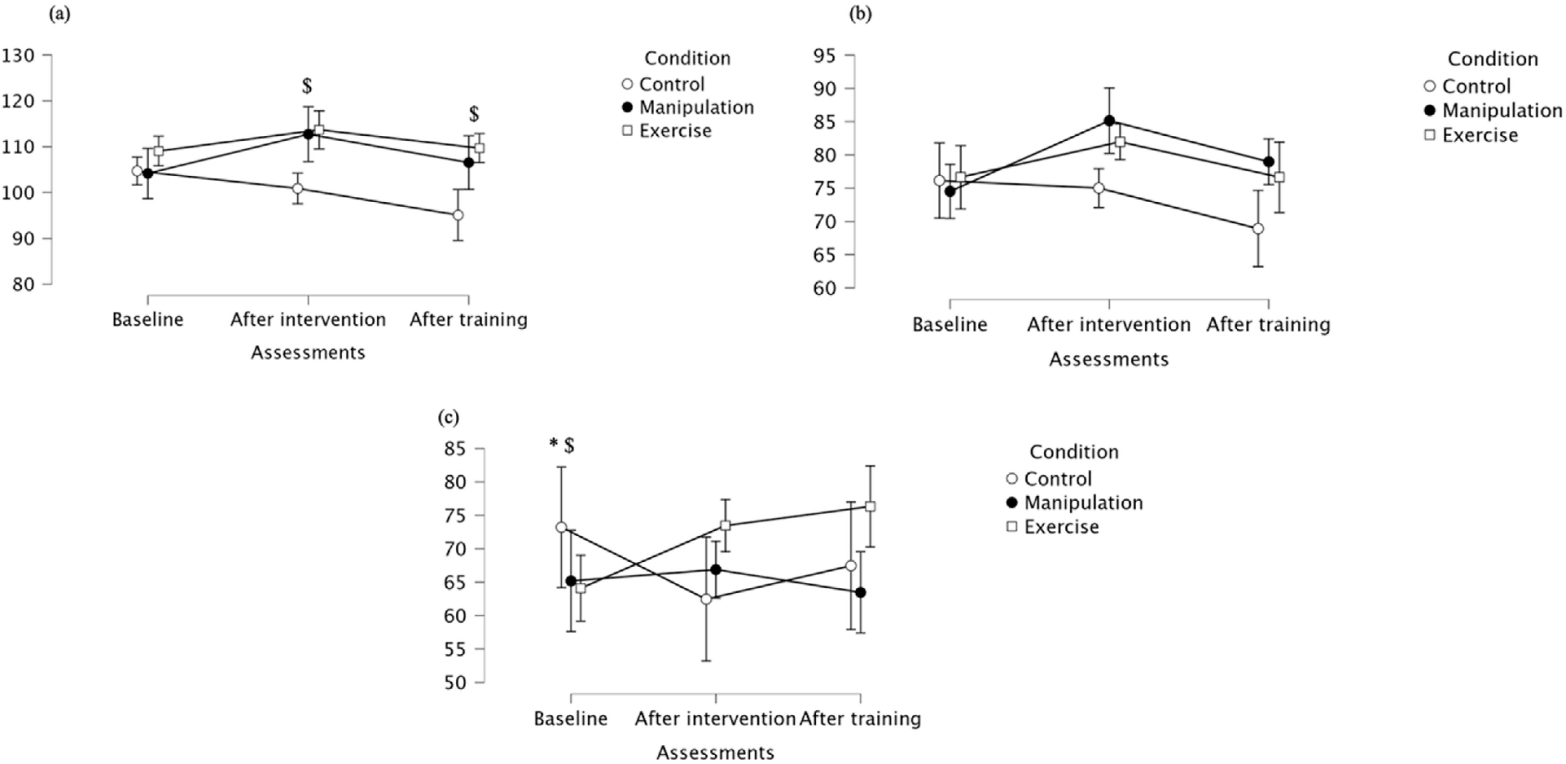
**(a)** Maximal, **(b)** mean, and **(c)** median activation of the vastus lateralis across groups. * Indicates significant differences between control and manipulation conditions; $ Indicates significant differences between control and exercise conditions.

Vastus lateralis maximal values showed significant effects for Assessment (p = 0.016) and Condition (p = 0.013), but no significant interaction (Table 1; Figure 5). Post-intervention and at follow-up, the Exercise group showed significantly higher maximal activation than Control. Descriptively, at baseline, maximal values were similar across groups. After the intervention, both the Exercise group and manipulation group increased activation, while the control group decreased. At follow-up, the Exercise group maintained higher activation compared to control, supporting the observed between-group differences. Regarding mean activation for Vastus lateralis, significant Assessment × Condition interaction (p = 0.011) was observed, as well as a significant main effect for Assessment (p = 0.015), but not for Condition (p = 0.203) (Table 1). Mean values were similar at baseline across groups. After the intervention, values increased notably in the Manipulation and Exercise groups, while the Control group remained stable. At follow-up, the Exercise group and manipulation group showed slight decreases but maintained higher means than the control group, supporting the observed interaction effect despite non-significant pairwise comparisons. Vastus lateralis median values revealed no significant main effects for Assessment (p = 0.877), Condition (p = 0.379), nor interaction (p = 0.189) (Table 1). Descriptively, baseline values were highest in the Control. Post-intervention, the Exercise group increased, while Control and Manipulation showed slight decreases. At follow-up, values remained stable in the control group, decreased in the manipulation group, and increased in the Exercise group. A single pairwise comparison (baseline: Exercise vs. Manipulation) was statistically significant (p = 0.041), though no consistent pattern was observed across timepoints. For detailed values, see Table 1.

**FIGURE 6:**
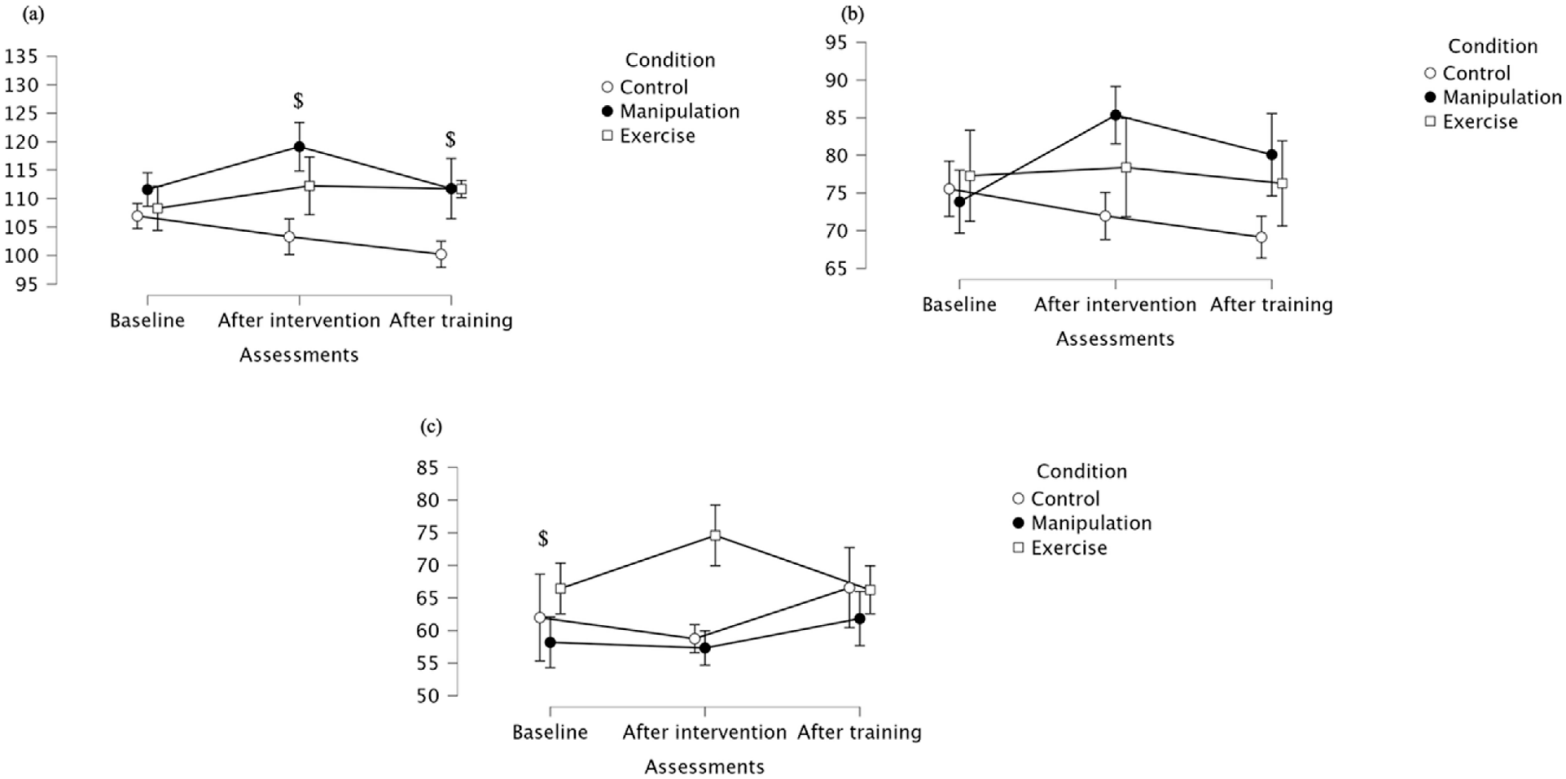
**(a)** Maximal; **(b)** Mean **(c)** Median of the vastus medialis across groups. $ Indicates significant differences between control and exercise conditions.

Vastus medialis maximal values showed significant effects for Assessment (p = 0.016) and Condition (p = 0.013), but no significant interaction (Table 1; Figure 6). Post-intervention and post-training, lunge exercises showed lower maximal activation than control. Descriptively, at baseline, maximal values were slightly higher in the manipulation group. After the intervention, the manipulation group maintained higher activation than Control and Exercise. The Exercise group consistently showed the lowest maximal values, particularly at follow-up, supporting the observed between-group differences. Vastus medialis mean values showed significant effects of Assessment (p = 0.015) and a significant interaction (p = 0.011), although pairwise comparisons did not reach significance (Table 1). Mean values were relatively similar across groups at baseline. After the intervention, the manipulation group presented a clear increase compared to Control and lunge. The Exercise group had the lowest mean values throughout the protocol, potentially explaining the interaction effect. Vastus medialis median values showed no significant effects, except for a baseline difference between Control and Exercise conditions (p = 0.041) (Table 1). Specifically, the Control group showed higher median values at baseline compared to the Exercise group. After the intervention, the Manipulation group had the lowest median value, while the Control group increased substantially. At follow-up, values across groups were comparable, reinforcing the absence of significant effects beyond the initial assessment. For detailed values, see Table 2.

**FIGURE 7:**
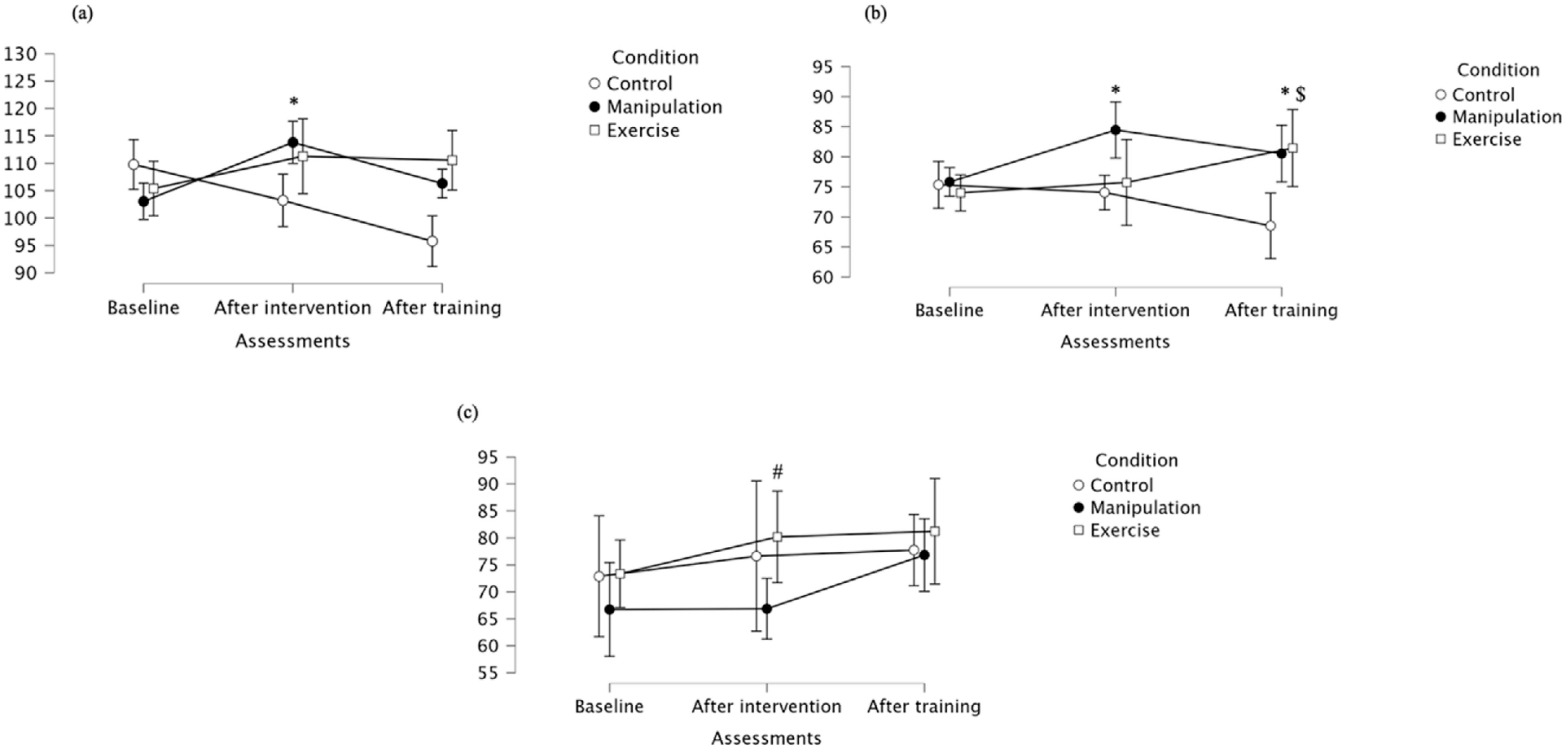
**(a)** Maximal; **(b)** Mean **(c)** Median of the rectus femoris across groups. * Indicates significant differences between control and manipulation conditions; # Indicates significant differences between manipulation and exercise conditions; $ Indicates significant differences between control and exercise conditions.

Rectus femoris maximal values showed a non-significant main effect of Assessment, but a significant interaction effect (p = 0.017) (Table 1). Post-intervention, the manipulation group exhibited significantly higher maximal activation than Control (Figure 7). Descriptively, baseline values were similar between groups. Following the intervention, the manipulation group increased markedly, while the Control group remained stable, and the Exercise group showed lower values at follow-up, supporting the interaction effect. Rectus femoris mean values showed no effect of Assessment, a significant Condition effect (p = 0.034), and no significant interaction (Table 1). Manipulation had significantly higher mean activation than Control post-intervention, and both Manipulation and Exercise groups showed higher activation than Control after training. Descriptively, at post-intervention, the Manipulation group showed the highest mean activation, while Control decreased. At follow-up, the Manipulation and Exercise groups had higher mean values compared to Control, in line with the statistical findings. Rectus femoris median activation showed no significant main or interaction effects (p > 0.05) (Table 1). However, pairwise comparisons revealed a significantly lower median activation in the Exercise group compared to the Manipulation group at post-intervention. After training, all groups showed similar median values, supporting the interpretation of a transient difference during the post-intervention phase. For detailed values, see Table 2.

Regarding Y-Balance Test Anterior performance, multivariate analysis showed a significant main effect of condition (Wilks’ Lambda = 0.149, F(2,7) = 20.036, p = 0.001, partial η^2^ = 0.851), indicating that intervention type significantly influenced performance (Table 3). The main effect of assessment was not significant (p = 0.146), though with a moderate effect size (partial η^2^ = 0.423). The interaction was also not significant (p = 0.103) but had a large effect size (partial η^2^ = 0.734), suggesting a potential trend over time depending on intervention. Descriptive statistics showed that performance generally improved from baseline to post-intervention in all groups, with a slight decrease post-training. Bonferroni-adjusted pairwise comparisons revealed that at baseline, Manipulation outperformed Control. After intervention and training, both Manipulation and Exercise groups performed significantly better than Control, with no differences between Manipulation and Exercise.

**TABLE 3:** Results of the repeated measures multivariate analysis (ANOVA repeated measures) for the effects of the intervention, assessment time, and their interaction on physical performance and proprioception variables.

Dependent variable	Effect	Wilks’ Lambda	F	df (hyp, error)	*p*	Partial η^2^
Y-balance test anterior	Intervention	2.562	2.562	(2,7)	0.146	0.42
	Assessment	0.149	20.036	(2,7)	**0.001**	0.85
	Intervention x Assessment	0.266	3.454	(4,5)	0.103	0.73
Y-balance posteromedial	Intervention	0.762	1.092	(2,7)	0.387	0.24
	Assessment	0.294	8.397	(2,7)	**0.014**	0.706
	Intervention x Assessment	0.153	6.940	(4,5)	**0.028**	0.847
Y-balance posterolateral	Intervention	0.474	3.885	(2,7)	0.073	0.526
	Assessment	0.289	8.618	(2,7)	**0.013**	0.711
	Intervention x Assessment	0.321	2.640	(4,5)	0.158	0.579
Standing broad jump	Intervention	0.743	1.208	(2,7)	0.354	0.257
	Assessment	0.786	0.952	(2,7)	0.431	0.214
	Intervention x Assessment	0.277	3.258	(4,5)	0.114	0.723
Proprioception 30°	Intervention	0.577	2.565	(2,7)	0.146	0.423
	Assessment	0.951	1.180	(2,7)	0.839	0.049
	Intervention x Assessment	0.448	1.543	(4,5)	0.319	0.552
Proprioception 45°	Intervention	0.751	1.162	(2,7)	0.367	0.249
	Assessment	0.921	0.299	(2,7)	0.751	0.079
	Intervention x Assessment	0.265	3.476	(4,5)	0.102	0.735
Proprioception 90°	Intervention	0.204	11.693	(2,6)	**0.009**	0.796
	Assessment	0.262	8.448	(2,6)	**0.018**	0.738
	Intervention x Assessment	0.165	5.064	(4,4)	0.073	0.835

Wilks’ Lambda, F-values, degrees of freedom, significance (p), and effect size (partial η^2^) are reported.

For Y-Balance Posteromedial, condition had a significant main effect (Wilks’ Lambda = 0.294, F(2,7) = 8.397, p = 0.014, partial η^2^ = 0.706) (Table 3). Assessment time was not significant (p = 0.387) but showed a moderate effect size (partial η^2^ = 0.238). The interaction was significant (Wilks’ Lambda = 0.153, F(4,5) = 6.940, p = 0.028, partial η^2^ = 0.847), indicating different changes over time across groups. Descriptive data clarify these findings: performance generally improved from baseline to post-intervention and remained stable post-training for all groups. Bonferroni-adjusted pairwise comparisons confirmed that both the Manipulation and Exercise groups significantly outperformed the Control group post-intervention and post-training, with no significant differences between the two active interventions.

For Y-Balance Posterolateral, condition had a significant main effect (Wilks’ Lambda = 0.289, F(2,7) = 8.618, p = 0.013, partial η^2^ = 0.711) (Table 3). Assessment time was not significant (p = 0.073), though with a moderate effect size (partial η^2^ = 0.526). The interaction was also not significant (p = 0.158) but showed a strong effect size (partial η^2^ = 0.679), suggesting meaningful group-dependent trends over time. Descriptive statistics support these findings. Performance generally increased from baseline to post-intervention for all groups, then slightly declined or stabilized post-training. Bonferroni-adjusted pairwise comparisons confirmed that both the Manipulation and Exercise groups demonstrated significant improvements over the Control group after intervention and training, with no significant differences between Manipulation and Exercise.

In the Standing Broad Jump test, no significant main effects were found for assessment (p = 0.354), condition (p = 0.431), or their interaction (p = 0.114), although the interaction effect size was relatively large (partial η^2^ = 0.723), suggesting potential group-specific trends over time (Table 3). Descriptive statistics reflect subtle differences across groups and time points. Despite these apparent trends, Bonferroni-adjusted pairwise comparisons revealed no statistically significant differences between groups at any time point.

For Proprioception at 30°, no significant main effects were found for assessment time (Wilks’ Lambda = 0.951, F(2,7) = 0.180, p = 0.839, partial η^2^ = 0.049), condition (Wilks’ Lambda = 0.577, F(2,7) = 2.565, p = 0.146, partial η^2^ = 0.423), or their interaction (Wilks’ Lambda = 0.448, F(4,5) = 1.543, p = 0.319, partial η^2^ = 0.552), although moderate-to-large effect sizes were observed, particularly for condition and interaction, suggesting possible group-related trends that did not reach statistical significance (Table 3). *Post hoc* Bonferroni-adjusted pairwise comparisons revealed a significant difference at baseline between the Manipulation and Exercise groups, indicating higher proprioceptive accuracy in the Manipulation group. No other pairwise differences were significant across time points or groups. Overall, although trends in the data suggest group-specific improvements, particularly in the Manipulation group, the results did not reach statistical significance over time or in interaction terms.

For Proprioception 45°, no significant effects were observed for assessment time (p = 0.367), condition (p = 0.751), or their interaction (p = 0.102), although the interaction effect size was moderate, suggesting a potential trend (Table 3). No significant pairwise differences were found at any time point. Although variations suggest small fluctuations across time points, particularly for the Manipulation group, none of the observed differences reached statistical significance, and the mean differences remained within a narrow range. Thus, no consistent or clinically meaningful changes were observed in proprioceptive performance at 45° for any group.

For Proprioception 90°, there was a significant main effect of assessment (p = 0.009, partial η^2^ = 0.796) and condition (p = 0.018, partial η^2^ = 0.738), indicating that both time and type of intervention had an impact on proprioceptive performance (Table 3). Although the interaction was not statistically significant (p = 0.073), it showed a strong effect size (partial η^2^ = 0.835), suggesting meaningful group-specific changes over time. Post hoc comparisons revealed that the Manipulation group performed significantly better than both the Control and Exercise groups post-intervention and post-training, while no differences were observed between Control and Exercise, nor between groups at baseline. These results indicate that all groups experienced some improvement, but the Manipulation group consistently achieved the highest proprioceptive scores post-intervention and post-training, supporting the observed significant effects and the interpretation of greater efficacy of Manipulation at 90° joint position sense.

## Discussion

The aim of the present study was twofold: (i) to investigate the effects of manual therapy through diagonal manipulation on gluteus medius muscle activation, and (ii) to examine the impact of the intervention on quadriceps femoris activation, another muscle essential for lower limb stability. The intervention consisted of a single session of diagonal manual therapy, with muscle activation measured using sEMG across multiple time points. Significant main and interaction effects were observed for gluteus medius maximal activation, with the Manipulation group demonstrating increases of approximately 17% in MVIC compared to baseline. Moreover, the intervention influenced the activation of other muscles critical to lower limb stability, particularly the vastus lateralis, where maximal, mean, and median activation values showed significant interaction effects with very large effect sizes. Significant interactions were also noted for the rectus femoris and vastus medialis muscles, indicating modulation of the quadriceps femoris group. Improvements were observed in dynamic balance, as indicated by significant time effects in all directions of the Y-Balance Test, along with a trend toward significance in proprioceptive acuity at 90° of hip flexion. These findings provide preliminary evidence supporting the effectiveness of diagonal manual therapy to enhance neuromuscular activation of key lower limb muscles and improve functional outcomes related to stability and proprioception.

The gluteus medius plays a key role in stabilizing the pelvis during dynamic lower limb movements such as gait, single-leg stance, and lateral displacement. The present findings support the effectiveness of diagonal manual therapy in enhancing gluteus medius activation, with significant effects observed for maximal activation levels. This suggests not only increased activation but also acute neuromuscular modulation over time. Although research specifically on diagonal manipulation is limited, related interventions have shown similar benefits. For example, proprioceptive neuromuscular facilitation (PNF) using diagonal two flexion significantly increased gluteus medius recruitment compared to other patterns (Miller et al., 2013; Youdas et al., 2015). Lumbopelvic manipulation improved EMG activity of the gluteus medius and vasti muscles, potentially benefiting athletes with patellofemoral pain (Lawrence et al., 2020). Hip-directed kinesio taping yielded immediate gains in postural stability and squat range of motion (Nardon et al., 2022), while ankle HVLA manipulations increased hip abductor strength in individuals with prior ankle sprains (Olewnik et al., 2022).

An interesting temporal pattern emerged in the TFL, where maximal activation decreased after both manipulation and exercise, then returned to near-baseline at follow-up. This may reflect a short-term neuromuscular inhibition or load redistribution, shifting effort away from the TFL toward stabilizers like the gluteus medius. Such a pattern may be beneficial in cases of TFL overactivity or faulty motor control (https://doi.org/10.1590/s1980-6574201900030011). In contrast, gluteus medius activation increased and remained elevated, indicating a more sustained facilitation. These divergent time courses suggest that muscle responses to intervention are not uniform, with some adaptations emerging immediately and others stabilizing over time. Clinically, this highlights the importance of timing assessments appropriately and considering delayed or transient effects. For practitioners, follow-up assessments may be essential to accurately capture the true impact of manual therapy on neuromuscular function, especially in dynamic tasks or rehabilitation planning.

Beyond the primary target muscle, the intervention also significantly influenced the activation of other key stabilizers, particularly the vastus lateralis, demonstrating large effects across maximal, mean, and median activation levels. This suggests a broad neuromuscular response, likely driven by the kinetic chain’s interconnected activation during diagonal manipulation. Additionally, modulation was observed in the quadriceps femoris group, including the rectus femoris and vastus medialis, indicating a possible synergistic effect that may enhance joint stabilization and functional capacity. This is consistent with motor control research showing that co-activation between hip and knee muscles supports postural control and load distribution (Lee, 2018; Grabara and Bieniec, 2023). Anatomically, the quadriceps femoris exhibits considerable variability, with extra muscle heads present in over 60% of individuals (Zakaria et al., 1997). Activation patterns among its components vary with the quadriceps angle, with increased lateral muscle activity observed at larger angles (Grindstaff et al., 2012). However, no preferential activation of quadriceps components was found during isometric exercises in females (Eymir et al., 2018). Furthermore, lumbopelvic manipulation did not produce immediate improvements in quadriceps force output in individuals with patellofemoral pain syndrome and was associated with decreased force over time, possibly due to muscle fatigue (Lalit et al., 2012). These findings emphasize the complex interplay between quadriceps anatomy, activation patterns, and the effects of manual interventions.

The study found significant improvements in dynamic balance measured by the Y-Balance Test, with large effect sizes suggesting meaningful gains in both intervention and control groups. Improvements across all directions likely reflect enhanced proprioceptive input and neuromuscular control induced by the manual therapy intervention (Bialosky et al., 2009). Manual therapy can stimulate mechanoreceptors in joint capsules, muscles, and surrounding soft tissues, increasing sensory feedback to the central nervous system. This heightened feedback improves neuromuscular coordination, dynamic postural stability, and motor control. Additionally, manual therapy may reduce pain and muscle tension, allowing for greater joint range of motion and more effective muscle activation patterns during dynamic tasks (Yutao, 2023). A trend toward better proprioceptive performance at 90° hip flexion indicates that diagonal manual therapy may positively affect joint position sense, a key factor in injury prevention and rehabilitation. This effect may be partly due to the hip position during diagonal manipulation, which likely targeted mechanoreceptors and neuromuscular pathways optimized for that specific joint angle, enhancing proprioceptive acuity at 90° (Riemann and Lephart, 2002). Research shows that joint position sense varies with knee angle, with better acuity at certain angles (Eymir et al., 2018). However, no significant changes were observed in SBJ performance. This may be explained by the specific demands of the SBJ, which primarily assesses explosive lower-limb power and coordination. The short duration of the intervention and its focus on neuromuscular control and proprioception may not have provided a sufficient stimulus to elicit measurable improvements in explosive jump ability. Furthermore, the healthy status of participants and inherent variability of SBJ measurements may have contributed to the lack of detectable change. Future research might consider longer or more targeted training protocols to impact this functional outcome. ACL-deficient patients display significant proprioceptive deficits compared to healthy controls, emphasizing the importance of injury status and knee angle in proprioceptive assessment and rehabilitation planning (Relph and Herrington, 2016).

The results have promising implications for clinical practice and athletic performance. Improvements in muscle activation and function suggest diagonal manual therapy as a valuable adjunct in rehabilitation for athletes prone to ankle sprains, those with neuromuscular deficits, or patients’ post-injury. By enhancing co-activation and motor control of key stabilizers, it may aid injury prevention and performance. Athletes with functional ankle instability show delayed muscle responses and reduced activity during diagonal landings, highlighting the need to assess postural stability and lower leg function in rehab (Kunugi et al., 2017). Core stability training benefits recovery in judokas (Cai, 2022), while manual therapy can reduce pain and improve gait by modulating spinal excitability (Courtney et al., 2011). A manual therapy protocol in field hockey players improved dynamic balance, ankle dorsiflexion, and lumbar flexibility, with effects lasting up to 4 weeks and outperforming proprioceptive neuromuscular facilitation in some aspects (Espí-López et al., 2018).

The findings of increased gluteus medius and vastus lateralis activation following diagonal manual therapy align with existing literature suggesting that manual interventions can acutely enhance neuromuscular function through spinal and supraspinal mechanisms (Keter et al., 2025). For example, manual therapy is known to modulate alpha motor neuron excitability and increase corticospinal excitability, potentially explaining the immediate gains in muscle activation observed here (Dishman et al., 2008). The diagonal application may further enhance these effects by recruiting multiple muscle groups across planes, resembling functional movement patterns and engaging broader motor pathways. Compared to traditional uniplanar manual techniques, the diagonal approach could therefore produce more extensive neuromuscular responses. This supports the theory that task-specific or movement-specific stimuli induce greater cortical representation and neuromuscular adaptation (Wühle et al., 2006). Furthermore, while prior studies have shown inconsistent results in quadriceps facilitation post-manipulation, our findings of significant vastus lateralis and rectus femoris modulation suggest that diagonal techniques may overcome some limitations observed in earlier studies, possibly due to the synergistic and multiplanar nature of the applied stimuli.

Despite the promising results, several limitations must be acknowledged. The small sample size reduces the generalizability of the findings and may limit the statistical power to detect more subtle effects. Additionally, the lack of long-term follow-up restricts conclusions about the durability of the observed neuromuscular changes. Variability in individual responses and potential learning effects across assessment sessions may have influenced some of the observed outcomes. Moreover, electromyographic assessments were limited to surface electrodes, which may not fully capture deep muscle activation or differentiate between synergistic and compensatory patterns. Importantly, the decision to examine only the acute effects of diagonal manual therapy was deliberate, as this study represents an initial step toward understanding the immediate neuromuscular responses induced by this specific technique. Given the scarcity of prior research focusing on diagonal manipulations, establishing short-term effects is crucial before implementing or recommending longer-term clinical applications. Acute responses also help identify potentially responsive muscles and mechanisms, guiding future protocols. Nonetheless, we acknowledge the importance of evaluating long-term outcomes, particularly for applications in injury prevention and rehabilitation, and recommend that future studies incorporate follow-up assessments to examine the sustainability and clinical relevance of these effects.

Building on these findings, future research should aim to evaluate the clinical utility of diagonal manual therapy in populations with functional deficits. Specifically, this approach may hold promise for individuals with chronic ankle sprains, functional ankle instability, or neuromuscular control impairments commonly observed in sports rehabilitation settings. Given the observed improvements in dynamic balance and activation of key stabilizers, integrating diagonal manual therapy into multimodal rehabilitation programs could enhance recovery of joint stability, reduce re-injury risk, and support return-to-play strategies. Longer-term randomized controlled trials are warranted to assess the sustainability of neuromuscular gains and their impact on functional performance and injury prevention in both athletic and clinical populations.

## Conclusion

The present study provides preliminary evidence that diagonal manual therapy effectively enhances neuromuscular activation of the gluteus medius, a key muscle for lower limb stability. Significant changes in maximal activation and interaction effects over time support its targeted facilitation. Additionally, the intervention influenced activation in other important muscles, such as the vastus lateralis and quadriceps femoris, indicating a broader neuromuscular response potentially beneficial for dynamic postural control. Improvements in dynamic balance and proprioception further suggest functional gains. These findings highlight diagonal manual therapy as a promising approach for injury prevention and rehabilitation, especially for individuals recovering from ankle sprains or athletes requiring enhanced lower limb stability.

## Data Availability

The original contributions presented in the study are included in the article/supplementary material, further inquiries can be directed to the corresponding author.
